# Impfeinstellung, Erwartungen und Impferfahrung von Immunsupprimierten bei COVID-19-Impfungen: Ergebnisse einer Längsschnittstudie

**DOI:** 10.1007/s00393-022-01213-5

**Published:** 2022-05-06

**Authors:** Frank Müller, Stephanie Heinemann, Eva Hummers, Eva Maria Noack, Gloria Heesen, Alexandra Dopfer-Jablonka, Marie Mikuteit, Jacqueline Niewolik, Sandra Steffens, Dominik Schröder

**Affiliations:** 1 Institut für Allgemeinmedizin, Universitätsmedizin Göttingen, Humboldtallee 38, 37073 Göttingen, Deutschland; 2 Klinik für Rheumatologie und Immunologie, Medizinische Hochschule Hannover, Hannover, Deutschland; 3 Deutsches Zentrum für Infektionsforschung, Hannover-Braunschweig, Deutschland

**Keywords:** Immunsuppression, Impfung, SARS-CoV‑2, Impfverweigerung, Immunmodulation, COVID-Impfung, Immunosuppression, Vaccination, SARS-CoV‑2, Vaccine hesitancy, Vaccination acceptance, Vaccine Uptake

## Abstract

**Hintergrund:**

Immunsupprimierte sind seltener geimpft, gleichzeitig profitieren sie im Hinblick auf die Nutzen-Risiko-Abschätzung deutlich von vielen Impfungen – auch bei den neuen Impfstoffen gegen SARS-CoV-2 (COVID-19). Bei der Entscheidung für eine Impfung sind Einstellungen, Erwartungen und Erfahrungen in Bezug auf bisherige Impfungen maßgeblich.

**Fragestellung:**

Welche Einstellungen haben immunsupprimierte Menschen gegenüber Impfungen allgemein und einer COVID-19 Impfung im Speziellen? Wie haben sie ihre COVID-19-Impfung erlebt?

**Material und Methoden:**

Im Rahmen der CoCo-Immun-Studie wurden im Frühjahr und Sommer 2021 (11.01.2021–07.11.2021) immunsupprimierte Teilnehmende zu 2 Zeitpunkten zu ihren Erwartungen an eine COVID-19-Impfung und zum Erleben der COVID-19-Impfung mit Fragebögen befragt. Zusätzlich wurden soziodemografische Daten, allgemeine Einstellungen gegenüber Impfungen sowie Erfahrungen mit bisherigen Impfungen erhoben. Die Auswertung erfolgte mittels deskriptiver und bivariater Statistik.

**Ergebnisse:**

Die 243 Befragten standen Impfungen überwiegend positiv und befürwortend gegenüber und erwarteten eine gute Verträglichkeit und Wirksamkeit. Frauen hatten weniger Vertrauen in die Sicherheit von Impfungen und häufiger Sorgen vor Impfreaktionen und -nebenwirkungen. Ältere Personen fühlten sich zum Zeitpunkt der Impfung besser informiert als jüngere. Personen, die über subjektive Nebenwirkungen bzw. Impfreaktionen bei vorangegangenen Impfungen berichteten, standen Impfungen und staatlichen Institutionen, die sie empfehlen, skeptischer gegenüber. Sie stimmten auch der Aussage „Rückblickend war die COVID-19-Impfung bisher harmlos für mich“ seltener zu.

**Diskussion:**

Die COVID-19-Impfungen wurden von den befragten Personen überwiegend positiv antizipiert. Die Alters- und Geschlechtsunterschiede in den Zustimmungswerten deuten jedoch darauf hin, dass es unterschiedliche Informationsbedürfnisse gibt, auf die es in Aufklärungsgesprächen und Impfkampagnen einzugehen gilt.

## Abstract (en)

**Background:**

Immunocompromised people are less likely to be vaccinated, despite an increased benefit of many vaccinations in terms of benefit-risk assessment, including the vaccines against SARS CoV-2 (COVID-19). Attitudes, expectations, and experiences with previous vaccinations influence the decision to get vaccinated.

**Objective:**

To explore the attitudes of immunocompromised people towards vaccinations in general and COVID-19 vaccination in particular and their experiences with COVID-19 vaccinations.

**Material and methods:**

As part of the CoCo Immune study, immunocompromised participants were surveyed in the spring and summer of 2021 (1 November 2021–7 September 2021) using questionnaires. Initially, they were asked about their expectations concerning a COVID-19 vaccination and followed up about their experience after COVID-19 vaccination. In addition, sociodemographic data, general attitudes toward vaccinations and experiences with previous vaccinations were collected. Analysis was performed using descriptive and bivariate statistics.

**Results:**

The 243 participants mostly approved vaccinations and expected the COVID-19 vaccination to be effective and well-tolerated. Women were more concerned about the safety of vaccinations and were more often worried about side effects. Older persons felt better informed than younger persons. Participants who reported subjective side effects of previous vaccinations were more skeptical about vaccinations as well as the government institutions that recommend vaccinations. They less often agreed with the statement “in retrospect, the COVID-19 vaccination has been harmless for me so far”.

**Discussion:**

The participants mostly expressed a positive attitude and anticipation towards COVID-19 vaccinations; however, the age and sex differences found suggest that there are different information needs which should be addressed when educating individuals about vaccinations or designing vaccination campaigns.

Immunsupprimierte profitieren im Hinblick auf eine COVID-19-Infektion in besonderem Maße von einer Impfung. Trotzdem sind sie – entsprechend einem allgemeinen Trend – seltener geimpft. Einstellungen gegenüber einer und Erwartungen an eine Impfung spielen dabei relevante Rollen bei der Impfentscheidung.

## Hintergrund und Fragestellung

Patienten mit Autoimmunerkrankungen oder anderen chronisch entzündlichen Erkrankungen haben ein erhöhtes Risiko für Infektionskrankheiten [11, 28]. Diese Vulnerabilität ist einerseits der Krankheit selbst, andererseits der Art und Dosierung immunsuppressiver Therapie zuzuschreiben [1, 31]. Studien zeigen für mehrere impfpräventable Infektionserkrankungen, dass Immunsupprimierte mit Autoimmunerkrankungen ein höheres Risiko für schwerwiegende Verläufe haben [23, 34]. Dies trifft ebenfalls auf die COVID-19-Erkrankung zu [9, 29]. Beschrieben ist ebenfalls, dass Infektionserkrankungen bestehende Autoimmunerkrankungen verschlechtern bzw. einen Schub auslösen können [19, 27]. Einen Schutz vor manchen infektiösen Erkrankungen bieten Impfungen. Während Lebendimpfstoffe bei Immunsupprimierten oft kontraindiziert sind, sind andere Impfungen sicher und oft besonders empfohlen [5, 32, 36]. Zwar können immunologische Impfantwort und somit die Wirksamkeit von Impfstoffen bei krankheitsbedingter oder iatrogener Beeinträchtigung des Immunsystems eingeschränkt sein [4, 17]. Nichtsdestotrotz profitieren Immunsupprimierte im Hinblick auf die Nutzen-Risiko-Abwägung von Impfungen besonders deutlich, was sich in den Empfehlungen der Ständigen Impfkommission widerspiegelt [36]. Trotzdem sind Immunsupprimierte generell [24, 36] seltener geimpft. Dies trifft auch auf spezifische Subgruppen, etwa Menschen mit chronisch entzündlichen Darmerkrankungen [37] oder Menschen mit entzündlichen rheumatischen Erkrankungen [20], zu. Eine zwischen Juni und September 2021 in Großbritannien durchgeführte Querschnittstudie mit über 50.000 Teilnehmern zeigte, dass 76,9 % der Patienten mit entzündlich rheumatologischen Erkrankungen nicht gegen COVID-19 geimpft waren, während die Vergleichsgruppe nicht-rheumatologisch Erkrankter eine Impfquote von 87,0 % aufwies [22]. Obwohl repräsentative epidemiologische Studien zur Durchimpfung Immunsupprimierter bisher fehlen, zeigte sich im vergangenen Jahr eine nicht unerhebliche Anzahl an Betroffenen einer Impfung skeptisch oder abwartend gegenüber: So beabsichtigten zwischen 15 % (USA [12]) und 20 % (Portugal [30]) Multiple-Sklerose-Erkrankter, 35,6 % von Menschen die unter chronisch entzündlichen Darmerkrankungen leiden (USA [8]), 14,7 % von Lebertransplantierten (Italien [10]) und 34,4 % von Menschen mit rheumatologischen Erkrankungen (Australien [21]), sich nicht gegen COVID-19 impfen zu lassen. Die vorliegende Untersuchung berichtet über Erwartungen und Einstellungen Immunsupprimierter zu Impfungen allgemein und insbesondere zur COVID-19-Impfung. Nach erfolgter Grundimmunisierung wurden Studienteilnehmer erneut befragt, wie sie die COVID-19-Impfung retrospektiv erlebten.

## Studiendesign und Untersuchungsmethoden

Die Auswertung basiert auf Daten der nichtinterventionellen Längsschnittstudie CoCo Immun, die Immunantwort, soziale Teilhabe und Impfeinstellung von Menschen mit einem hohen Risiko für einen schwerwiegenden Verlauf einer COVID-Erkrankung untersucht. Die vorliegende Studie beruht auf der Auswertung einer Teilstichprobe, nämlich Menschen mit medikamentöser Immunsuppression aufgrund von Autoimmunerkrankungen oder nach Organtransplantation. Andere im Rahmen der Studie rekrutierte Subgruppen von hämatologisch-onkologischen Patienten unter Tumorbehandlung sowie Menschen über 80 Jahren (ohne medikamentöse Immunsuppression) wurden für die Auswertung exkludiert. Ein Protokoll beschreibt das Studienprocedere (Protokoll wird nach erfolgter Veröffentlichung referenziert).

### Rekrutierung

Immunsupprimierte Personen wurden im Frühjahr 2021 über Aushänge in Impfzentren, bei Haus- und Fachärzten sowie im Rahmen von Medienberichterstattungen in Südniedersachsen und der Region Hannover über die Möglichkeit einer Teilnahme informiert. Teilnehmende konnten bis 30 Tage nach zweiter Impfung eingeschlossen werden. Einschlusskriterien für die Gruppe der Immunsupprimiertenkohorte waren (a) die Einnahme einer systemisch wirksamen immunsuppressiven Medikation (also eine bestehende iatrogene Immunsuppression durch einem Wirkstoff der ATC-Gruppe [Anatomical Therapeutic Chemical] L04) zum Zeitpunkt des Einschlusses *oder* systemische Kortikosteroidtherapie mit einem Prednisolonäquivalent von mindestens 2,5 mg/Tag und (b) Absicht, sich gegen COVID-19 impfen zu lassen *oder* eine bereits erfolgte, höchstens 30 Tage zurückliegende zweite Impfung und (c) vollendetes 18. Lebensjahr und (d) bestehende Einwilligungsfähigkeit und abgegebene schriftliche Einwilligung zur Studienteilnahme. Ausschlusskriterien waren (a) fehlende systemische, pausierte oder nur lokal wirksame immunsuppressive Therapie, (b) fehlende Absicht, eine COVID-19-Impfung zu erhalten, *oder* zurückliegende zweite Impfung liegt länger als 30 Tage zurück, (c) Minderjährigkeit, (d) nicht bestehende Einwilligungsfähigkeit oder fehlende Einwilligung.

### Fragebögen

Mittels Fragebögen wurden alle Teilnehmenden zum Studieneinschluss (Baseline) sowie 1 Monat nach abgeschlossener Grundimmunisierung (Follow-up) befragt. Grundimmunisierung meint dabei den Erhalt von 2 Dosen eines zu diesem Zeitpunkt in Deutschland zugelassenen Impfstoffes bzw. einer Dosis des Impfstoffs von Johnson und Johnson (Ad26.COV2.S, Janssen-Cilag/Johnson und Johnson), bei dem zunächst eine Einzeldosis als abgeschlossene Grundimmunisierung galt.

Mit dem Baseline-Fragebogen wurden dabei die immunsuppressive Medikation samt Dosierung sowie die die Medikation begründende Erkrankung erfragt. Ferner wurden soziodemografische Daten und das Vorliegen anderer Vorerkrankungen erhoben. Um die Einstellung gegenüber Impfungen im Allgemeinen zu erheben und mit anderen Stichproben zu vergleichen, wurden Items aus dem Infektionsschutzsurvey der Bundeszentrale für Gesundheitliche Aufklärung (BZgA) von 2018 einbezogen [16]. Zusätzliche Fragen erfassten bisherige Erfahrungen mit Impfungen und etwaige Impfreaktionen bzw. -Nebenwirkungen. Diese wurden ergänzt um Items, die spezifisch Erwartungen, Ängste, Befürchtungen gegenüber den COVID-19-Impfungen widerspiegeln. Die Items wurden in einem diskursiven Prozess gemeinsam mit mehreren, nicht am Forschungsprojekt beteiligten Wissenschaftlern entwickelt und anschließend zusätzlich mit 2 immunsupprimierten Laien diskutiert. In einem Pretest wurde der finalisierte Fragebogen 10 am Forschungsprozess unbeteiligten Personen vorgelegt, um ihn auf Verständlichkeit zu prüfen und den Zeitbedarf zum Ausfüllen zu erheben. Die Personen brauchten dabei etwa 5 min, um den Baseline-Fragebogen bzw. 2,5 min um den Follow-up-Fragebogen auszufüllen.

Alle im Fragebogen erhobenen Items stellen dabei Aussagen dar. Die Teilnehmenden konnten auf einer 5‑stufigen Likert-Skala angeben, wie sehr sie den Aussagen zustimmten (stimme voll zu – stimme eher zu – teils-teils – stimme eher nicht zu – stimme nicht zu) und hatten ferner die Möglichkeit, „weiß nicht/nicht zutreffend“ anzukreuzen.

Im Follow-up-Fragebogen wurden die erhaltenen Impfstoffe und das Datum der Impfungen sowie Erfahrungen und Einschätzungen zur COVID-19-Impfung erfragt. Item- und Skalenentwicklung erfolgten analog zum Baseline-Fragebogen.

### Datenhaltung und Auswertung

Die Fragebögen wurden von den Teilnehmenden für die Baseline-Erhebung direkt nach dem Studieneinschluss ausgefüllt bzw. abgegeben und für das Follow-up 4 Wochen nach Abschluss der Grundimmunisierung mittels vorfrankierten Versandboxen an die Studienzentrale geschickt.

**Figure 1:**
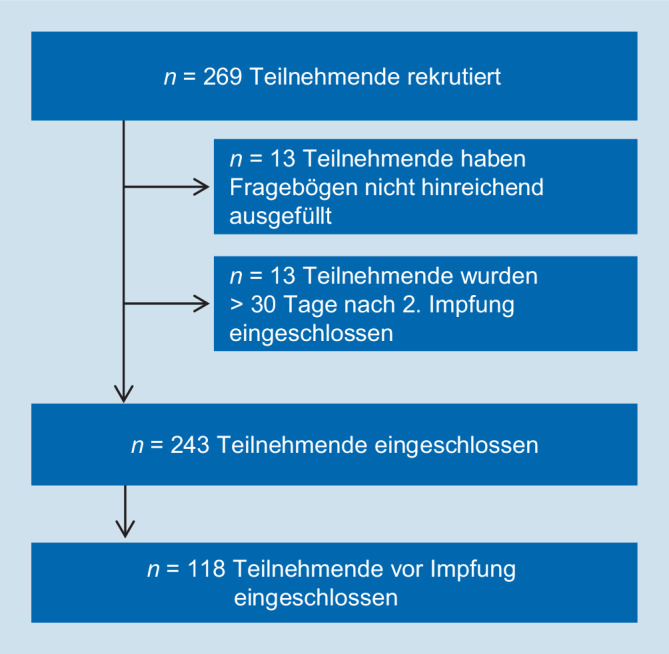

Um eine Erinnerungsverzerrung (Recall Bias) zu vermeiden, wurden die Fragebogenitems, die die Erwartung an die COVID-19-Impfung widerspiegeln, nur in der Subgruppe derjenigen ausgewertet, die zum Studieneinschluss noch nicht gegen COVID-19 geimpft waren (vgl. Abb. 1).

Daten wurden in das EvaSys-Umfragesystem (EvaSys GmbH, Lüneburg, Deutschland) eingelesen, Fehlerkennungen korrigiert und direkt in SPSS 27 (IBM, Armonk, NY, USA) exportiert, womit auch die weitere Auswertung erfolgte. Statistische Auswertungen umfassten deskriptive Statistik mit Darstellung von absoluten und relativen Häufigkeiten, Median und Interquartilsabstand (IQR). Unterschiede zwischen Geschlechtern und anderen kategorialen Variablen wurden unter Verwendung des Fisher’s Exact-Test bei 2 × 2-Kontingenztafeln und bei größeren Kontingenztafeln mit dem Chi-Quadrat-Test (bei erwarteten Zellenwerten ≥ 5) oder dem Fisher-Freeman-Halton Exact-Test (bei erwarteten Zellenwerten < 5) beschrieben. Bei der teststatistischen Auswertung des Einflusses von Geschlecht, Alter und Angabe von Impfreaktionen und -nebenwirkungen bei vorangegangenen Impfungen auf Punktescores der jeweiligen Items fand der Mann-Whitney-*U*-Tests bzw. Kruskal-Wallis-Test-Anwendung. Für teststatistische Auswertungen wurde die Antwortkategorie „weiß nicht/nicht zutreffend“ exkludiert und auf „fehlend“ gesetzt. Fälle mit fehlenden Werten („missing values“) wurden von der jeweiligen Auswertung exkludiert. *p*-Werte < 0,05 wurden als signifikant gewertet.

### Ethik

Die Studie erhielt Ethikvoten der Universitätsmedizin Göttingen (29/3/21) und der Medizinischen Hochschule Hannover (8973_BO_K_2020). Die Studie ist im Deutschen Register für Klinische Studien registriert (DRKS00023972).

## Ergebnisse

Von 269 initial in die Studie eingeschlossenen Teilnehmenden wurden 26 von der weiteren Auswertung ausgeschlossen, da sie entweder Fragebögen gar nicht abgegeben hatten („loss to follow up“), über 50 % der untersuchten Items nicht beantwortet hatten oder zu spät eingeschlossen wurden. Von den verbleibenden 243 Teilnehmenden wurden 118 vor der ersten Impfung eingeschlossen (vgl. Abb. 1).

Befragte füllten den Baseline-Fragebogen (vor Impfung bis 30 Tage nach abgeschlossener Grundimmunisierung) zwischen dem 11.01. und 19.08.2021 und den Follow-up-Fragebogen (1 Monat nach Grundimmunisierung) zwischen dem 04.03. und 07.09.2021 aus. Zwischen Follow-up-Fragebogen und Abschluss der Grundimmunisierung lagen im Median 30 Tage (IQR 28–33).

Die 243 eingeschlossenen Teilnehmenden waren überwiegend weiblich (70,8 %) und im Median 54 (IQR 44–60) Jahre alt. Im Hinblick auf das Alter zeigten sich keine signifikanten Unterschiede zwischen den Geschlechtern. Frauen waren häufiger alleinlebend und wiesen häufiger rheumatologische Erkrankungen auf. Näheres zeigt Tab. 1.

**Table 1:** 

–		Biologisches Geschlecht^d^		
		Männlich *n* = 69 (29,2)	Weiblich *n* = 167 (70,8)	–
		*n* (%)	*n* (%)	*p*^j^
Alter^a^	Median (IQR)	54 (41–62)	52 (44–59)	0,467
	18–34	10 (14,7)	21 (12,6)	0,141
	35–64	44 (61,8)	123 (73,7)	
	65+	16 (23,5)	23 (13,8)	
Schulabschluss^b^	Keinen/Volksschule	1 (1,5)	1 (0,6)	0,001
	Hauptschulabschluss	8 (12,3)	8 (5,0)	
	Realschulabschluss/POS (Mittlere Reife)	9 (13,8)	57 (35,4)	
	Abitur/Fachhochschulreife	41 (63,1)	93 (57,8)	
	Sonstiger	6 (9,2)	2 (1,2)	
Wohnort^c^	< 5000 Einwohner	28 (43,8)	55 (36,4)	0,446
	5000– < 20.000 Einwohner	9 (14,1)	31 (20,5)	
	20.000– < 100.000 Einwohner	7 (10,9)	24 (15,9)	
	Über 100.000 Einwohner	20 (31,3)	41 (27,2)	
Migrationshintergrund^e^	–	4 (6,2)	4 (2,5)	0,186
Haushalt^f^	Mit Kindern	17 (24,6)	32 (20,0)	0,432
	Alleinlebend	9 (13,0)	41 (25,6)	0,034
Grad der Behinderung^g^	Keinen	29 (42,0)	57 (34,3)	0,395
	20–49 %	14 (20,3)	37 (22,3)	
	50–74 %	18 (26,1)	59 (35,5)	
	75–100 %	8 (11,6)	13 (7,8)	
COVID-Impfschema^a^	2‑mal mRNA	56 (82,4)	124 (75,2)	0,485
	2‑mal AstraZeneca	3 (4,4)	11 (6,7)	
	Heterolog	9 (13,2)	23 (13,9)	
	Sonstige/unbekannt	0 (0)	7 (4,2)	
Immunsuppressive Medikation^h^	Konventionelle Immunsuppression	31 (44,9)	80 (47,9)	0,677
	Kortikosteroide	19 (27,9)	52 (31,1)	0,628
	TNF-Inhibitoren	14 (20,3)	31 (18,6)	0,759
	Andere Biologicals	11 (15,9)	15 (9,0)	0,120
	Sonstige	3 (4,3)	15 (9,0)	0,222
Erkrankung^i^	Rheumatoide Arthritis, Morbus Bechterew, Sjögren-Syndrom	21 (30,4)	87 (52,1)	0,002
	Psoriasis und Psoriasisarthritis	15 (21,7)	30 (18,0)	0,502
	Chronisch entzündliche Darmerkrankungen	15 (21,7)	26 (15,6)	0,255
	Multiple Sklerose	10 (14,5)	18 (10,8)	0,422
	Z. n. Organtransplantation	7 (10,1)	7 (4,2)	0,078
	Sonstige Erkrankung	7 (10,1)	20 (12,0)	0,688

^a^ Fehlend *n* = 2

^b^ Fehlend *n* = 10

^c^ Fehlend *n* = 23

^d^ Fehlend *n* = 7

^e^ Gem. Definition der Bundesagentur für Arbeit, fehlend *n* = 13

^f^ Fehlend *n* = 8

^g^ Fehlend *n* = 1

^h^ Mehrfachnennung bei Kombinationstherapie möglich, konventionelle Immunsuppression umfasst Methotrexat, Azathioprin, Leflunomid, Mycophenolat-Mofetil, Tacrolimus, Everolimus; Kortikoide umfassen Prednison/Prednisolon, Hydrocortison; TNF-Inhibitoren umfassen Etanercept, Adalimumab, Certolizumab, Golimumab, Infliximab; andere Biologicals umfassen Tocilizumab, Ustekinumab, Vedolizumab, Secukinumab, Guselkumab, Sonstige umfassen Hydroxychloroquin, Fingolimod, Upadacitinib

^i^ Mehrfachnennung bei Multimorbidität möglich

^j^ Alter (numerisch): Mann-Whitney-*U*, alle anderen: Chi-Quadrat- bzw. Fisher’s Exact-Test/Fisher-Freeman-Halton Exact-Test bei Zellenwerten < 5

### Generelle Einstellung gegenüber Impfungen (BZgA Infektionsschutzsurveys)

**Figure 2:**
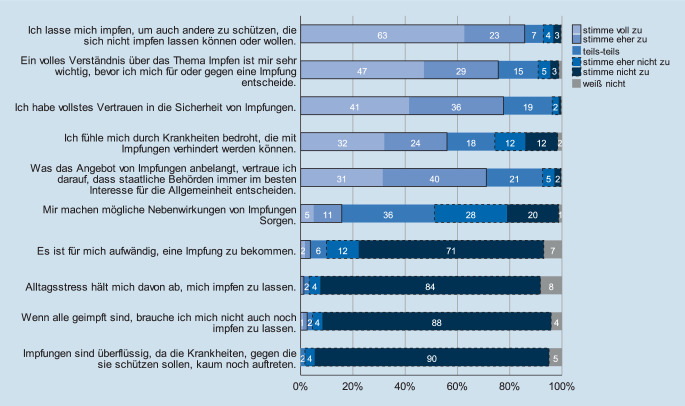

Die Mehrheit der Befragten standen Impfungen befürwortend (81,9 %) oder eher befürwortend gegenüber (14,4 %). Keiner der Befragten äußerte sich „ablehnend“ oder „eher ablehnend“ gegenüber Impfungen. Altruistische Motive standen bei der Entscheidung für Impfungen im Vordergrund (86 % voll zustimmend oder eher zustimmend). Hohe Zustimmungswerte erhielten ferner die Aussage, sich vor einer Impfung ein volles Verständnis schaffen zu wollen, sowie das generelle Vertrauen in die Sicherheit von Impfungen (s. auch Abb. 2). Frauen hatten jedoch etwas weniger Vertrauen in die Sicherheit von Impfungen (4,8 % stimmen nicht oder eher nicht zu vs. 0 % bei männlichen Teilnehmern, *p* = 0,001) und hatten häufiger Sorgen vor Impfreaktionen bzw. -nebenwirkungen (19,3 % stimmen zu oder eher zu vs. 5,7 % bei männlichen Teilnehmern, *p* < 0,001). Ältere Teilnehmende tendierten ferner dazu, der Aussage, sich ein volles Verständnis über Impfungen verschaffen zu wollen, zuzustimmen (*p* < 0,001).

Befragte, die über deutliche Impfreaktionen bzw. Nebenwirkungen in Zusammenhang mit vorangegangenen Impfungen berichteten (s. unten), hatten weniger Vertrauen in die Sicherheit von Impfungen (stimme nicht zu/eher nicht zu 9,4 % vs. 2,6 %, *p* = 0,002), stimmten seltener zu, dass staatliche Behörden im besten Interesse der Allgemeinheit entscheiden (stimme nicht zu/eher nicht zu 16,2 % vs. 4,4 %, *p* = 0,033) und äußerten häufiger Sorgen gegenüber möglichen Impfreaktionen bzw. -nebenwirkungen (stimme voll zu/eher zu 18,8 % vs. 10,7 %, *p* = 0,006).

### Bisherige Erfahrungen mit Impfungen

Auf die Frage „Hatten Sie jemals Nebenwirkungen in Zusammenhang mit Impfungen“ äußerten 21,6 % „gar keine“ und 44,8 % „kaum welche bemerkt“. Deutliche Impfreaktionen bzw. -nebenwirkungen („ja, sehr stark“ und „stark“) gaben insgesamt 13,2 % der Befragten an. Starke Impfreaktionen bzw. -nebenwirkungen wurden signifikant häufiger von Frauen angegeben (16,2 % vs. 4,4 %, *p* = 0,016). Die Angabe zur Stärke von Reaktionen bzw. Nebenwirkungen war unabhängig vom Alter der Befragten (*p* = 0,250). Diejenigen, die starke oder sehr starke Reaktionen oder Nebenwirkungen angaben, äußerten im Vergleich zu denjenigen, die über keine oder kaum Reaktionen oder Nebenwirkungen berichteten, auch weniger Vertrauen in die Sicherheit von Impfungen („stimme nicht zu“/„eher nicht zu“ 9,7 % vs. 2,6 %, *p* = 0,002) zu haben. Menschen mit Impfreaktionen oder -nebenwirkungen bei vorherigen Impfungen hatten zudem weniger Vertrauen darin, dass staatliche Behörden im besten Interesse der Allgemeinheit entscheiden („stimme nicht zu“/„eher nicht zu“ 16,2 % vs. 4,4 %, *p* = 0,033) und hatten häufiger Sorgen vor möglichen Impfreaktionen oder -nebenwirkungen („stimme voll zu“/„eher zu“ 19,4 % vs. 10,8 % *p* = 0,006).

Häufigste Impfreaktion bzw. -nebenwirkungen bisheriger Impfungen waren Schmerzen an der Einstichstelle (53,5 %), Fieber/grippale Symptome (21,8 %), allgemeine längere Schwäche (14,8 %), Hautrötung/Entzündung/Ausschlag (13,2 %), sonstige Reaktionen/Nebenwirkungen (13,2 %). Seltener wurden Juckreiz (5,3 %), allergische Reaktion (4,5 %), Atemnot (0,8 %), Energieverlust/Ängste/Depression (2,1 %) sowie Probleme mit Hören/Sehen (0,4 %) genannt. Signifikant häufiger berichteten Frauen von allgemeiner länger dauernder Schwäche (19,2 % vs. 4,3 % *p* = 0,004).

### Erwartungen gegenüber der COVID-19-Impfung

**Figure 3:**
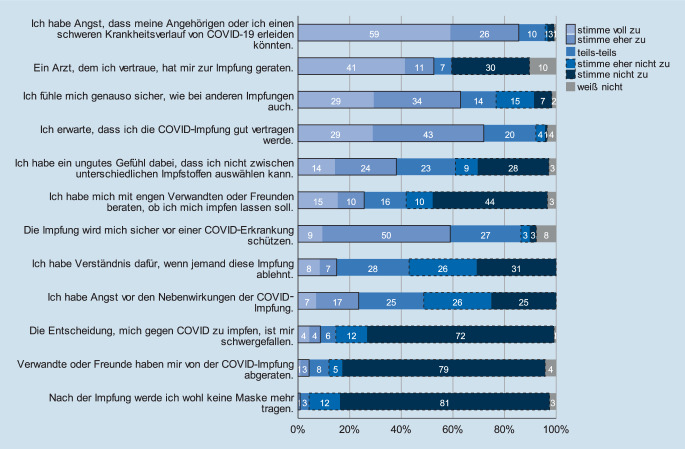

Erwartungen gegenüber der COVID-19-Impfung wurden anhand der Subgruppe (*n* = 118) ausgewertet, die den Baseline-Fragebogen vor einer COVID-19-Impfung ausfüllte. Eine große Mehrheit äußerte Angst, dass sie selbst oder Angehörige einen schweren Krankheitsverlauf erleiden könnten (85 % stimme voll zu/eher zu), jedoch auch die Zuversicht, dass die Impfung gut vertragen werde (72 % stimme voll zu/eher zu). Etwa zwei Drittel sahen die Impfung als genauso sicher wie andere Impfungen an (63 % stimme voll zu/eher zu). Deutlich heterogener zeigte sich die Einschätzung über ein Unbehagen, nicht zwischen Impfstoffen auswählen zu können (zustimmend 38 %, nicht zustimmend 37 %). Die überwiegende Mehrheit hatte kein Verständnis für andere Menschen, die die COVID-19-Impfung ablehnen (57 %) (vgl. Abb. 3).

Nur wenige Befragten gaben an, dass ihnen die Entscheidung zur Impfung schwergefallen sei (8 % stimme voll zu/eher zu). Etwas mehr als die Hälfte (52 %) der Befragten habe ein Arzt zur Impfung geraten (stimme voll zu/eher zu), nur eine Minderheit (25 % stimme voll zu/eher zu) habe sich mit engen Verwandten oder Freunden besprochen.

Unter den weiblichen Befragten stimmten 5,3 % nicht oder eher nicht zu, dass sie erwarten, die Impfung gut zu vertragen (vs. 0 % bei männlichen Teilnehmern, *p* = 0,004). Frauen hatten auch eher Angst vor Impfreaktionen bzw. -nebenwirkungen einer COVID-19-Impfung (29,9 % stimme voll zu/eher zu vs. 5,8 % bei männlichen Teilnehmern, *p* < 0,001) und stimmten seltener der Aussage „Ich fühle mich genauso sicher wie bei anderen Impfungen auch“ zu (35,5 % stimme eher nicht zu/nicht zu vs. 14,3 % bei männlichen Teilnehmern, *p* = 0,002). Auch das „ungute Gefühl, […] nicht zwischen unterschiedlichen Impfstoffen“ auswählen zu können, wurde eher von den befragten Frauen als von den Männern bejaht (47,0 % stimme zu/eher zu vs. 18,8 % männliche Teilnehmer, *p* = 0,024).

Ältere Befragte tendierten dazu, „eher nicht“ oder „nicht“ der Aussage zuzustimmen, Verständnis für Menschen aufzubringen, die eine COVID-19-Impfung ablehnen (*p* = 0,002).

Unabhängig davon, ob Impfreaktionen bzw. -Nebenwirkungen bei vorangegangenen Impfungen erlebt wurden oder nicht, haben Teilnehmende zumeist ähnliche Erwartungen gegenüber einer COVID-19-Impfung berichtet. Einzig die erwartete Wirksamkeit unterschied sich deutlich: Die Aussage „Die Impfung wird mich sicher vor einer COVID-19-Erkrankung schützen“ bewerteten 45,5 % der Befragten mit vorangegangenen Impfreaktionen bzw. -nebenwirkungen mit „stimme eher nicht zu/nicht zu“ vs. 2,4 % derjenigen, die über keine Reaktionen/Nebenwirkungen berichteten (*p* = 0,002).

Teilnehmende mit rheumatologischen Erkrankungen äußerten häufiger, Angst vor Nebenwirkungen einer COVID-19-Impfung (30,0 % stimme zu/stimme eher zu vs. 18,5 %, *p* = 0,019) zu haben, aber seltener Angst davor, einen schwerwiegenden COVID-19-Verlauf erleiden zu können (76,9 % stimme voll zu/eher zu vs. 93,8 %, *p* = 0,023) als nicht-rheumatologisch Erkrankte. Eingeschlossene Teilnehmende mit Psoriasis stimmten häufiger der Aussage zu, ein Unbehagen zu empfinden, nicht zwischen Impfstoffen auswählen zu können (60 % stimme voll zu/eher zu vs. 44,5 % unter Nicht-Psoriasis-Erkrankten, *p* = 0,030).

### Einschätzung nach der Impfung

**Figure 4:**
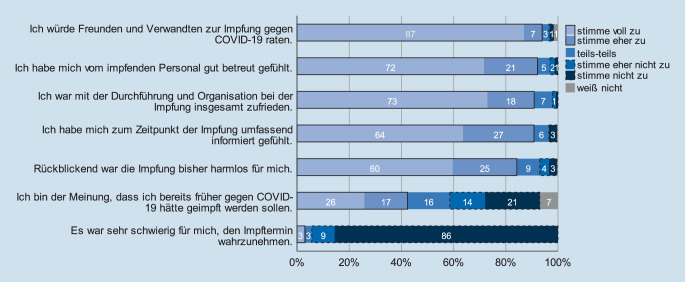

Die rückblickende Einschätzung der Impfung 1 Monat nach erfolgter Grundimmunisierung war durchweg positiv: 94 % würden Freunden und Verwandten zur Impfung gegen COVID-19 raten (stimme voll zu/eher zu), es bestand eine große Zufriedenheit mit der Betreuung während der Impfung (93 % stimme voll zu/eher zu) sowie Zustimmung zu einem Gefühl umfassender Informiertheit (jeweils 91 % stimme voll zu/eher zu); 85 % der Befragten äußerten Zustimmung zu der Aussage „Rückblickend war die Impfung bisher harmlos für mich“. Sehr heterogen war die Einschätzung darüber, ob man bereits früher hätte geimpft werden sollen (vgl. Abb. 4).

Teilnehmerinnen waren im Hinblick auf die Aussage, ob die Impfung harmlos für sie gewesen sei, zurückhaltender als männliche Teilnehmer (82,1 % vs. 88,0 %, *p* = 0,010). Sonst zeigten sich keine signifikanten Unterschiede zwischen den Geschlechtern.

Ein deutlicher altersspezifischer Trend zeigt sich bei der Frage nach der Informiertheit zum Zeitpunkt der Impfung: Je älter Teilnehmer waren, desto eher stimmten sie dieser Aussage zu (*p* < 0,001). In ähnlicher Weise wurde die Betreuung durch die impfenden Personen von älteren Befragten meist positiver eingeschätzt (*p* = 0,041).

Auffällig war, dass diejenigen Befragten, die angaben, bei vorherigen Impfungen bereits unter starken Impfreaktionen bzw. -Nebenwirkungen gelitten zu haben, der Aussage „Rückblickend war die Impfung bisher harmlos für mich“ seltener zustimmten als diejenigen, die keine solche Erfahrungen mit vorherigen Impfungen berichteten (stimme nicht zu/eher nicht zu 25,0 % vs. 3,5 %, *p* = 0,001). Etwas geringer, aber dennoch signifikant fiel der Unterschied bei der Aussage „Ich bin der Meinung, dass ich bereits früher gegen COVID-19 hätte geimpft werden sollen“ zwischen diesen Gruppen aus: Teilnehmer, die über keine Impfreaktionen oder -nebenwirkungen bei vorherigen Impfungen berichteten, stimmten in 48,9 % Fälle der Aussage voll oder eher zu vs. 33,3 % bei Befragten mit früheren Impfreaktionen bzw. -nebenwirkungen (*p* = 0,043). Weitere signifikante Unterschiede zwischen diesen Gruppen sowie zwischen unterschiedlichen Erkrankungsgruppen, etwa hinsichtlich der empfundenen Betreuung während der Impfung oder zum Informationsstand bei der Impfung, konnten nicht gezeigt werden.

## Diskussion

Gegen COVID-19 haben sich die in Europa zugelassenen Impfstoffe als hochwirksam erwiesen: Sie schützen bis zum gegenwärtigen Zeitpunkt in sehr hohem Maße vor schwerwiegenden und tödlichen Verläufen der Erkrankung und sind sicher in ihrer Anwendung [2, 14, 33, 35]. Dies kann auch bei den bisher aufgetretenen Virusvarianten beobachtet werden [25, 26]. Trotz dieser Tatsache sind in Deutschland gegenwärtig weiterhin 23,4 % der Bevölkerung, für die es zugelassene Impfstoffe gäbe, nicht gegen COVID-19 geimpft [6].

Die vorliegende Studie untersuchte immunsupprimierte Menschen, die bei Studieneinschluss beabsichtigt hatten, sich impfen zu lassen oder sogar bereits eine Impfung gegen COVID-19 erhalten hatten. Die Rationale der Studie lag darin zu erfahren, welche Erwartungen und Einstellungen sie gegenüber Impfungen allgemein und der COVID-19-Impfung im Besonderen hatten.

Die Auswertung zeigt auf den ersten Blick in vielen Teilen erwartbare oder auch sozial erwünschte Ergebnisse: Impfungen wurden allgemein als positiv erachtet, Angst vor Impfreaktionen/-nebenwirkungen äußerte nur ein geringer Anteil der Befragten, und es bestand ein großes Vertrauen in Institutionen und die Sicherheit von Impfstoffen. Erfreulich – und möglicherweise auch ein Argument in der Aufklärung von Unentschlossenen – ist, dass die allermeisten Immunsupprimierten nach erfolgter COVID-19-Impfung diese als harmlos einschätzten. Eine große Heterogenität in den Antworten bestand insbesondere in der Frage, ob man früher hätte geimpft werden sollen, und hinsichtlich eines Unbehagens, nicht zwischen unterschiedlichen Impfstoffen auswählen zu können.

Fast ein Viertel der Befragten äußerten Ängste gegenüber „Nebenwirkungen der COVID-Impfung“, und 5 % der Befragten erwarteten, die COVID-Impfung nicht oder eher nicht gut vertragen zu werden. Im Gegensatz dazu zeigen Anwendungsbeobachtungen, dass schwerwiegende Nebenwirkungen wie thromboembolische Ereignisse (21 bis 75 Fälle auf 1 Mio. verimpfte Dosen) bei den gegenwärtig kaum mehr verimpften Vektorimpfstoffen bzw. Myokarditiden (2 bis 3 Fälle auf 1 Mio. verimpfte Dosen) bei den mRNA-basierten Impfstoffen ebenso wie schwere allergische Reaktionen sehr selten sind [7]. Häufig sind hingegen Impfreaktionen wie Fieber, grippeähnliche Symptome, Schmerzen an der Einstichstelle etc., die aber nur kurzzeitig persistieren und in der Regel keiner weiteren Behandlung bedürfen [7]. In einer dänischen Studie zeigten sich bei rheumatologischen Patienten nach Impfung mit einem mRNA-Impfstoff geringfügig häufiger bestimmte Impfreaktionen als in einer gesunden Kontrollkohorte [3]. Über diese erwartbaren und für Betroffene mitunter lästigen, aber in der Regel harmlosen Reaktionen sollte hinreichend aufgeklärt sowie Maßnahmen zur Linderung von Beschwerden und Stärkung der Selbstwirksamkeit sollten besprochen werden. Letztere Studie zeigte nämlich ebenfalls, dass rheumatologisch erkrankte Patienten zur Linderung der Impfreaktionen seltener auf Antipyretika zurückgriffen [3].

Etwa ein halbes Jahr vor unserer Befragung – und vor der Zulassung wirksamer COVID-19-Impfstoffe – führte die BZgA eine Erhebung mit über 5000 Personen durch [15]: Im Vergleich zu dieser Erhebung war die Rate an Impfbefürwortern in unserer Stichprobe um etwa 17 Prozentpunkte höher, und das Vertrauen unserer Teilnehmenden in die Sicherheit von Impfungen lag um 14 Prozentpunkte höher. Ebenso widersprachen mehr Teilnehmende in unserer immunsupprimierten Stichprobe der Aussage, dass Impfungen überflüssig seien (+10 %).

In der Analyse spezifischer Subgruppen – nach Alter, Geschlecht und ob immunsupprimierte Impfkandidaten über Impfreaktionen bzw. -nebenwirkungen bei vorangegangenen Impfungen berichteten – zeigte sich ein weit weniger einheitliches Bild. So zeigen sich deutliche Bewertungsunterschiede zwischen den Geschlechtern, die nahelegen, dass immunsupprimierte Männer mit der bevorstehenden COVID-19-Impfung deutlich weniger haderten: Sie empfanden die COVID-19-Impfung häufiger als sicher, weniger nebenwirkungsbehaftet und hatten seltener ein Problem damit, nicht zwischen unterschiedlichen Impfstoffen auswählen zu können. Diese Tendenz ist nicht neu und konnte kürzlich in einer Metaanalyse bestätigt werden [38]. Die vorliegende Studie zeigte dabei, dass diese Tendenz sich auch prinzipiell bei Impfwilligen reproduzieren lässt. Nach der Impfung äußerten Männer häufiger, dass die Impfung für sie harmlos gewesen sei. Dies lässt mutmaßen, dass zwischen den Geschlechtern unterschiedliche Informationsbedürfnisse bestehen, welches die Ergebnisse einer spanischen Studie bestätigen [13]. Mit der Frage, ob bzw. wie Frauen und Männer über Impfungen unterschiedlich aufgeklärt werden, gibt es nach unserem Wissen bisher keine hinreichende wissenschaftliche Auseinandersetzung. Weitere Forschung könnte dafür sorgen, auf Informationsbedürfnisse besser einzugehen und sie sowohl in Aufklärungsgesprächen als auch in Informationskampagnen besser zu adressieren.

Ebenfalls auffällig waren die Einschätzungen bei Befragten, die über vorangegangene Nebenwirkungen anderer Impfungen berichteten. Hierbei muss stets beachtet werden, dass es sich um die subjektive antizipierte Schwere von „Nebenwirkungen“ handelt und dass es sich bei diesen sog. Nebenwirkungen in den allermeisten Fällen offensichtlich eher um normale Impfreaktionen handelte als um echte oder gar schwere Nebenwirkungen. Impfreaktionen bzw. -nebenwirkungen wurden dabei nicht ärztlich validiert. Wir hatten auch wenig naheliegende und selten bis gar nicht beschriebene Nebenwirkungen „allgemeine längere Schwäche“ oder „Energieverlust/Ängste/Depression“ erfragt, um auch der Impfung zugeschriebene, aber mit großer Wahrscheinlichkeit davon unabhängige oder durch negative Erwartungen ausgelöste Symptome zu erheben. Teilnehmer mit Impfreaktionen bzw. -nebenwirkungen bei anderen Impfungen hatten zwar kein vermindertes Vertrauen in die Sicherheit der COVID-19-Impfung ex ante, jedoch in Impfungen allgemein und in das Handeln der Behörden im Hinblick auf Impfempfehlungen. Diese Gruppe ging häufiger davon aus, dass die Schutzwirkung vor einer COVID-19-Erkrankung geringer sei (dies wurde zu einem Zeitpunkt im Sommer 2021 erhoben, wo die COVID-19-Inzidenz gering und Impfdurchbrüche bei den zirkulierenden Varianten Alpha und Delta wenig prävalent waren). Aus diesen Ergebnissen lässt sich ebenfalls ableiten, dass gerade Menschen, deren empfundene Nebenwirkungen schwer objektivierbar und nach gängigen Kriterien nicht als schwerwiegend einzustufen sind, häufiger mit der Entscheidung über eine Impfung hadern – und ebenfalls andere Informationsbedürfnisse haben.

Zuletzt ist noch ein weiteres Ergebnis bemerkenswert: Ältere Immunsupprimierte fühlten sich bei der Impfung besser informiert und besser betreut. Dies kann damit zusammenhängen, dass ihre eigene Nutzen-Risiko-Kalkulation stark zugunsten der Impfung ausfiel und die Erfahrungen ihrer Peers mit COVID-Erkrankungen einen entsprechenden Eindruck hinterlassen haben. Auch andere Präventionsangebote (jenseits von Impfungen) scheinen bei älteren Zielgruppen besser „anzukommen“ [18]. Es ist denkbar, dass die bisherigen Aufklärungsmethoden und Informationen auf eine immer älter werdende Zielpopulation zugeschnitten sind. Grundlegendere Forschung zu effektiver und altersspezifischer Vermittlung von Präventionsangeboten inklusive einer Erforschung der Informationsbedürfnisse sowie der geeigneten Methoden dieser Informationsvermittlung wären wünschenswert.

Die Studie unterliegt Limitationen, die bei der Interpretation der Ergebnisse beachtet werden sollten. So wurden lediglich Personen eingeschlossen, die beabsichtigten, sich gegen COVID-19 impfen zu lassen. Der strukturelle Ausschluss von Impfgegnern könnte die Ergebnisse verzerren. Eine Stärke der Studie liegt in der rekrutierten Stichprobe, die Immunsupprimierte aus der täglichen Primärversorgung gut abbildet. Gleichzeitig kann ein Rekrutierungsbias durch die Art der Ansprache von freiwilligen Teilnehmenden nicht ausgeschlossen werden. Für eine weitere Untersuchung wäre eine vergleichbare Befragung von impfunwilligen Immunsupprimierten oder Immunsupprimierten, die initial mit einer COVID-19-Impfung haderten, wünschenswert.

## Schlussfolgerungen

Die Ergebnisse dieser Befragung verdeutlichen eine hohe Akzeptanz gegenüber Impfungen allgemein als auch der COVID-19-Impfung unter impfwilligen Immunsupprimierten. Hierbei zeigen sich Alters- und Geschlechtsunterschiede, die bei zukünftigen Impfkampagnen und der Gesundheitskommunikation mitberücksichtigt werden sollten.

## Fazit für die Praxis

- Unter impfwilligen Immunsupprimierten kann eine hohe Befürwortung von Impfungen allgemein und spezifisch der COVID-19-Impfung beobachtet werden.
- Männer standen der bevorstehenden COVID-19-Impfung positiver gegenüber und empfanden die COVID-19-Impfung als sicherer im Vergleich zu Frauen.
- Unterschiede bezüglich der Impfakzeptanz sollten bei zukünftigen Impfkampagnen und der Gesundheitskommunikation mitberücksichtigt werden.
